# The First Meeting of the Executive Committee of the Ninth International Medical Congress

**Published:** 1885-10

**Authors:** 


THE FIRST MEETING OF THE EXECUTIVE COMMITTEE OF THE NINTH INTERNATIONAL MEDICAL CONGRESS.

The newly-created Executive Committee, consisting of the officers of the preliminary organization of the congress, including the presidents of the sections, held its first meeting in New York on September 24th, effected its organization and assumed charge of the remaining preliminary work. *

Henry H. Smith, of Philadelphia, was elected chairman, and F. S. Dennis, of New York, Secretary *pro tem.* Subsequently Professor Dennis was elected Associate Secretary-General, and R. J. Dunglison, of Philadelphia, was elected Chairman of the Finance Committee.

The following resolution was passed:

*Resolved,* That this Executive Committee enters upon the management of the affairs of the Ninth International Medical Congress, with the understanding that, in accordance with Rule No. 10, its powers are not restricted except by the rules and regulations adopted Sept. 3, 1885, by the Committee of Arrangements appointed by the American Medical Association in April, 1885; and that the actions of this Executive Committee, are final, not being subject to revision, amendment or alteration by either the Committee of Arrangements or the American Medical Association.

The Rule 10, alluded to in this resolution, is as follows:

"10. The President of the Congress, the Secretary-General, the Treasurer, the Chairman of the Finance Committee, and the Presidents of the Sections, shall together constitute an Executive Committee of the Congress, which Committee shall direct the business of the Congress, shall authorize all expenditures for the immediate purposes of the Congress, shall supervise and audit the accounts of the Treasurer, and shall fill all vacancies in the offices of the Congress and the Sections. This Committee shall have power to add to its membership, but the total number of members shall not exceed thirty. A number equal to one-third of the members of the Committee shall consitute a quorum for the transaction of business."

After the transaction of some routine business the Executive Committee adjourned subject to the call of its Chairman.

